# Lithium orotate: A superior option for lithium therapy?

**DOI:** 10.1002/brb3.2262

**Published:** 2021-07-01

**Authors:** Anthony G. Pacholko, Lane K. Bekar

**Affiliations:** ^1^ Department of Anatomy Physiology and Pharmacology College of Medicine University of Saskatchewan Saskatoon Saskatchewan Canada

**Keywords:** increased therapeutic window, Lithium Toxicity, Maintenance Therapy, Mania, Mood Stabilizer, Pharmacokinetics

## Abstract

Bipolar disorder (BD) poses a significant public health concern, with roughly one‐quarter of sufferers attempting suicide. BD is characterized by manic and depressive mood cycles, the recurrence of which can be effectively curtailed through lithium therapy. Unfortunately, the most frequently employed lithium salt, lithium carbonate (Li_2_CO_3_), is associated with a host of adverse health outcomes following chronic use: these unwanted effects range from relatively minor inconveniences (e.g., polydipsia and polyuria) to potentially major complications (e.g., hypothyroidism and/or renal impairment). As these undesirable effects can limit patient compliance, an alternative lithium compound with a lesser toxicity profile would dramatically improve treatment efficacy and outcomes. Lithium orotate (LiC_5_H_3_N_2_O_4_; henceforth referred to as LiOr), a compound largely abandoned since the late 1970s, may represent such an alternative. LiOr is proposed to cross the blood–brain barrier and enter cells more readily than Li_2_CO_3_, which will theoretically allow for reduced dosage requirements and ameliorated toxicity concerns. This review addresses the controversial history of LiOr, complete with discussions of experimental and clinical efficacy, putative mechanisms of action, adverse effects, and its potential future in therapy.

## Graphical abstract

Li_2_CO_3_ is an efficacious therapeutic option in BD, but a troublesome side effect profile limits patient compliance. LiOr may represent an alternative form of lithium that can enter the brain more readily than Li_2_CO_3_, which would theoretically allow for maintenance of efficacy at reduced dosages, thereby mitigating non‐compliance concerns by minimizing the incidence of dose‐dependent side effects.

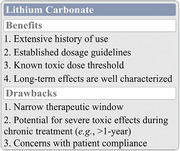

## 1 INTRODUCTION

Bipolar Disorder (BD) afflicts roughly 3% of North Americans and poses a significant public health concern, with nearly 25% of sufferers attempting suicide (Hilty et al., 2006; Prien & Potter, 1990). BD is characterized by manic and depressive states, each replete with its own set of problematic symptoms, for example, impulsive when manic, suicidal when depressive (Culpepper, 2014). Lithium salts have been used for more than half a century to address the psychiatric manifestations of BD, and while other medications have since been introduced, namely antipsychotics, lithium remains a key therapeutic option (Culpepper, 2014). Of the presently prescribed lithium salts, lithium carbonate (Li_2_CO_3_) is the most commonly administered, and arguably holds a position among the most effective medications for the prevention of mood‐episode recurrence and maintenance of the relatively stable euthymic phase of the disorder (Machado‐Vieira et al., 2009; Malhi et al., 2020; Won & Kim, 2017).

While the use of lithium has declined (Karanti et al., 2016; Tondo et al., 2019), largely in favor of increased prescription of antipsychotics (Malhi et al., 2020; Zivanovic, 2017), evidence exists which suggests that such a reduction in employment may be somewhat ill‐advised, particularly within the context of maintenance therapy (Malhi et al., 2020; Severus et al., 2014; Zivanovic, 2017). The results of a meta‐analysis conducted by Miura et al. (2014) found that of the 17 treatment options assessed in 33 randomized controlled trials, only lithium and quetiapine prevented manic‐ and depressive‐episode relapse or recurrence when used in monotherapy. Notably, the data for lithium were derived from trials with non‐enriched designs, which was not the case for quetiapine (Nolen, 2015). Thus, while antipsychotics represent an efficacious alternative, lithium maintains a role in the prevention of manic‐ and/or depressive‐episode recurrence, either alone or in combination with other medications.

Unfortunately, Li_2_CO_3_‐based therapy is not without issues. First and foremost, Li_2_CO_3_, much like other approved lithium compounds, has a narrow therapeutic window coupled with a side‐effect profile that ranges from inconvenient (e.g., nausea) to potentially life‐threatening (e.g., renal dysfunction). Unsurprisingly, patient non‐compliance with Li_2_CO_3_ treatment is a frequently encountered issue (Öhlund et al., 2018). Patients that respond well to Li_2_CO_3_ are faced with a conundrum: are the side‐effects worth the alleviated symptoms? Thus, it would be of enormous benefit if a similarly effective medication could be identified that displays an expanded therapeutic window and a less severe side‐effect profile.

Intriguingly, such a medication may already exist in the form of a different lithium complex: lithium orotate (LiC_5_H_3_N_2_O_4_; henceforth referred to as LiOr). LiOr is most notable for its early use and advocacy by the controversial Hans Nieper in the 1970s, who proposed that orotic acid was a superior carrier compound that could more readily transport inorganic ions across biological membranes (Nieper, 1970, 1973). In support of this, a study by Kling et al. (1978) found that LiOr resulted in brain lithium concentrations three‐fold higher than what were observed for equivalent doses of Li_2_CO_3_. While the concentration of lithium within the serum fell over the course of 24 h in mice treated with either compound, only those injected with LiOr displayed a progressive increase in brain lithium levels (Kling et al., 1978), which suggests an alternative, and perhaps superior, set of mechanisms underpinning its cellular influx and/or efflux. Also of note, orotic acid itself may confer antioxidant benefit beyond its role as a mineral carrier (Aonuma et al., 1969; Hassani et al., 2019; Loffler et al., 2016). Unfortunately, concerns were raised in 1979 about the potential for increased renal toxicity relative to Li_2_CO_3_ (Smith & Schou, 1979), though given the results noted by Kling et al. (1978), it is likely that the concentrations of LiOr used were far too high. These concerns effectively curtailed LiOr‐related research for decades.

At present, LiOr maintains nutraceutical status, and is thus available over‐the‐counter without prescription. Unsurprisingly, this ease of access has contributed to its adoption by self‐medicating populations, alternative health practitioners, and some physicians, thereby necessitating in‐depth examinations, which are largely absent, into its efficacy, tolerability and safety.

Considering the lack of literature surrounding LiOr, this review aims to call attention to (1) the history of Li_2_CO_3_ use, (2) why Li_2_CO_3_ rose to prominence, (3) the issues associated with Li_2_CO_3_‐based therapy, (4) why research into LiOr stagnated, and (5) how future explorations into LiOr as an option in the management of BD are needed.

## 2 WHAT IS BIPOLAR DISORDER?

### 2.1 Prevalence and symptomatology

BD is a relatively prevalent mood disorder with an incidence rate exceeding 3% in North America. BD poses a significant health risk, with roughly one quarter of patients attempting suicide (Hilty et al., 2006; Prien & Potter, 1990). BD is an umbrella term referring to a category of chronic illnesses characterized by depressive/major depressive and manic/hypomanic mood cycles, with the severity of each cycle varying according to the specific class; for example, BD I, BD II, and cyclothymia. (Culpepper, 2014). Depressive states are characterized by feelings of persistent sadness, anhedonia, sleep disturbance, and fatigue, whereas mania and hypomania are noted for euphoria, loss of inhibition, impaired decision‐making, and/or reduced need for sleep (Association AP, 2013).

### 2.2 Etiopathogenesis

The underpinnings of these conditions have not been fully elucidated, though theories regarding their etiopathogenesis, such as circadian clock dysfunction (Takaesu, 2018), catecholamine excess/deficiency/dysfunction (Ashok et al., 2017; Kurita, 2016), serotonin dysregulation (Lee et al., 2012; Mahmood & Silverstone, 2001), aberrant glycogen synthase kinase‐3β (GSK3β) (Jope & Roh, 2006; Jope, 2011; Muneer, 2017; Yu & Greenberg, 2016), inositol monophosphatase (IMPase) activity (Yu & Greenberg, 2016), neuroinflammation (Hamdani et al., 2013; Muneer, 2016; Roda et al., 2015), and loss/lack of neurotrophic factors (Li et al., 2014; Roda et al., 2015), have been proposed. It is worth noting that nearly all the above‐mentioned phenomena can be argued to be downstream consequences of dysregulated GSK3β and IMPase activity.

## 3 THE ROLE OF LITHIUM THERAPY: A BRIEF OVERVIEW

**FIGURE 1:**
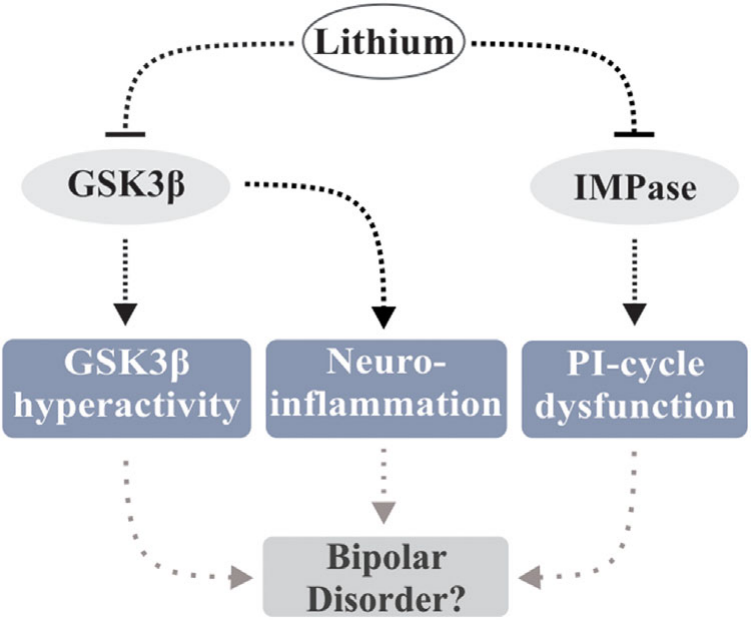
Putative mechanisms underpinning the therapeutic efficacy of lithium salts in BD. GSK3β hyperactivity, PI‐cycle dysfunction and neuroinflammation have been implicated in BD etiopathogenesis. Lithium may alleviate expression of BD symptoms through amelioration of these processes, likely though mechanisms involving displacement of magnesium (thereby limiting activity) from the catalytic cores of GSK3β and IMPase

Lithium salts, brought to the forefront as a therapeutic option by John Cade in 1949 (Cole, 2013) and Samuel Gershon in 1960 (Gershon & Yuwiler, 1960), have a well‐established role in BD treatment, most notably the control of mania and the prevention of mood‐disturbance recurrences. Lithium is thought to contribute to the management of affective disorders through a variety of mechanisms, with the antagonism of GSK3β and IMPase (Yu & Greenberg, 2016) standing as perhaps the most prominent one (Figure 1). Inhibition of these enzymes is mediated through the displacement of the cofactor magnesium; lithium and magnesium share similar ionic radii, allowing lithium to act as a competitive inhibitor for the binding of the magnesium ion (Mg^2+^) at the catalytic core of each protein (Brown & Tracy, 2013; Lu et al., 2012; Ryves & Harwood, 2001). Also, a growing body of evidence has linked neuroinflammation to a host of affective disorders, BD included. As lithium may demonstrate anti‐inflammatory capacities, attenuation of neuroinflammation may present an additional mechanism by which lithium addresses the symptoms of BD. While the above discussions are intriguing, it should be noted that the mechanisms of action for lithium in the alleviation of BD symptoms are far from being conclusively identified.

### 3.1 Inhibition of GSK3β

Lithium salts are known inhibitors of GSK3β (Ryves & Harwood, 2001). This antagonistic function has been proposed as a central mechanism by which lithium contributes to the alleviation of BD symptoms. Aberrant GSK3β activity has been linked to the disruption of the circadian clock (Cole, 2013; Levenson & Frank, 2011; Paul et al., 2012; Takaesu, 2018), impaired neurogenesis and hippocampal function and/or reduced volume (Elvsåshagen et al., 2013; Hartberg et al., 2015; Otten & Meeter, 2015), and decreased expression of neurotrophic factors (Grimes & Jope, 2001; Li et al., 2014; Soares et al., 2016; Yasuda et al., 2009), all of which are pathological processes believed to be associated with BD.

Interestingly, transgenic mice with reduced GSK3β activity mimic behaviors observed in chronic lithium treatment (Cole, 2013; O'Brien et al., 2004), while those that overexpress GSK3β exhibit mania‐like hyperactivity (Prickaerts et al., 2006); in humans, BD patients display reduced inhibitory serine phosphorylation of GSK3β, which result in elevated enzymatic activity (Polter et al., 2010).

Also, chronic treatment of BD with lithium rescues the expression of brain‐derived neurotrophic factor (BDNF) (De Sousa et al., 2011), which is often reported to be reduced in the brains of BD patients (Li et al., 2014; Soares et al., 2016). This may potentially be attributable to excessive GSK3β activity, and the resultant suppression of BDNF mRNA transcription that occurs through the inhibition of cAMP‐response element‐binding protein (Grimes & Jope, 2001). Given the reciprocal regulatory relationship between GSK3β and BDNF (Fiol et al., 1994; Grimes & Jope, 2001; Gupta et al., 2014), it is possible that lithium upregulates BDNF levels through the inhibition of GSK3β; application of GSK3β antagonists increases transcription of the BDNF gene (Yasuda et al., 2009).

These findings implicate dysregulation of GSK3β in BD pathogenesis, and appear to establish the inhibition of its activity as a likely mediator of lithium's therapeutic actions.

### 3.2 Inhibition of IMPase

Myo‐inositol is a substrate used for the synthesis of phosphatidylinositol phosphates (Shi et al., 2006), which are signaling molecules involved in a diverse array of processes, including cell growth, cell proliferation, apoptosis, neuronal transmission, and the actions of insulin, among others (Toker, 2002).

Altered levels of brain inositol have been observed in BD patients (Kato et al., 1991; Shimon et al., 1997; Yildiz et al., 2001). In addition, phosphatidylinositol‐4, 5‐bisphosphate levels (Soares & Mallinger, 1996), and protein kinase C activity (Friedman et al., 1993) are found to be elevated in BD, suggesting aberrations within the phosphoinositol cycle (PI‐cycle).

Lithium inhibits IMPase activity (Forlenza et al., 2014; Patel et al., 2002) through the displacement of Mg^2+^ at catalytic sites (Patel et al., 2002) resident within the enzyme. Intracellular concentrations of myo‐inositol are reduced in rodent models following the administration of lithium salts (Allison & Stewart, 1971; Allison et al., 1976; Berridge et al., 1982). As aberrant inositol levels and dysfunction of the PI‐cycle have been noted in BD patients (Davanzo et al., 2001; Kato et al., 1991; Shimon et al., 1997; Silverstone et al., 2002; Yildiz et al., 2001), it is possible that lithium derives part of its therapeutic efficacy in BD from the inhibition of IMPase and subsequent normalization of the PI‐cycle. In support of this, normalization of myo‐inositol and phosphomonoester concencentrations in chronic‐use BD patients has been observed (Silverstone et al., 2002).

It is important to note that the consequences of lithium‐mediated IMPase inhibition are less well characterized than GSK3β antagonism within the context of BD. Far more research is required before any definitive conclusions can be made regarding the “inositol depletion” hypothesis (Berridge et al., 1989) of lithium's therapeutic efficacy (Raghu et al., 2019).

### 3.3 Attenuation of neuroinflammation

BD patients often present with comorbid inflammatory conditions (Perugi et al., 2015; Rosenblat & McIntyre, 2015) and/or elevated expression of proinflammatory cytokines, such as interleukin‐4 (IL‐4), interleukin‐6 (IL‐6), interleukin‐1β (IL‐1β) and tumor necrosis factor‐α (TNFα) (Barbosa et al., 2014; Brietzke et al., 2009; Brietzke et al., 2009). Cytokines are signaling molecules involved in either the potentiation or attenuation of inflammation, among numerous other functions. They are generally classified as either pro‐ or anti‐inflammatory, which allows the cytokine expression profile, at a select point in time, to represent the current inflammatory state of a given tissue/system. Interestingly, both bipolar parents and their symptomatic/at‐risk asymptomatic children display a gene profile characteristic of elevated immune‐inflammatory signaling (Padmos et al., 2008), which suggests a potential role for neuroinflammation in BD etiology as well as pathogenesis.

Intriguingly, the expression of the proinflammatory cytokines, for example, IL‐4, IL‐6, IL‐1β, and TNFα, is often rescued/normalized in previously medication‐naive BD patients following chronic treatment with lithium (Boufidou et al., 2004; Knijff et al., 2007; Rapaport et al., 1999).

The anti‐inflammatory effects (Dong et al., 2014; Nassar & Azab, 2014) of lithium may be reliant on mechanisms involving GSK3β antagonism; lithium inhibits GSK3β (Ryves & Harwood, 2001), which is involved in the induction of pro‐inflammatory microglial responses to inflammatory stimuli (Yuskaitis & Jope, 2009). It is therefore possible that aspects of lithium's therapeutic effects may be elicited through anti‐inflammatory mechanisms involving GSK3β inhibition‐dependent attenuation of microglial proinflammatory responses.

### 3.4 Where does lithium orotate enter the picture?

As previously discussed, GSK3β and IMPase are potentially at the root of much of BD symptomatology. Given that both enzymes display intracellular localization, it is possible that a lithium formulation that more readily crosses biological membranes, or displays increased intracellular residency‐time, would result in greater control of BD symptoms. LiOr has been proposed to do just that, with evidence from animal studies suggesting that not only does LiOr yield higher brain concentrations of Li^+^ than Li_2_CO_3_, but also that the levels of Li^+^ progressively build over the course of 24 h (Kling et al., 1978). If inhibition of GSK3β and IMPase are truly the mechanisms through which lithium exerts its therapeutic actions, then these observed elevations in brain Li^+^ may enable the employment of LiOr at concentrations that are substantially lower than those currently prescribed for Li_2_CO_3_. Furthermore, the sustained increase in brain Li^+^ over 24 h may allow for a more robust and consistent control of symptoms between doses.

## 4 THE HISTORY OF LITHIUM SALTS AND THEIR USES

### 4.1 Discovery

Lithium was named by chemist Johan August Arfvedson, who isolated the element from petalite procured from the soils of the island of Uto. This intriguing substance was so named after the Greek word *lithos*, which translates to “from stone.” Not surprisingly, lithium is most typically found concentrated in salars (saline basins), clay beds, and bedrock mines throughout South America, North America, Australia, and China. Lithium is so abundant in these areas that it frequently makes its way into both food and water supplies. Intriguingly, the absence/decreased levels of lithium in drinking water are associated with increased incidence of a host of harmful mood‐related conditions (Schrauzer & Shrestha, 1990; Schrauzer et al., 1992; Sugawara et al., 2013).

### 4.2 Early applications of lithium salts in psychiatry

The use of lithium in psychiatry has roots in the mid‐1800s. Some credit this early interest in lithium to Alfred Baring Garrod, who used lithium in the treatment of gout (Garrod, 1860), as uric acid was often considered to be at the root of a host of diseases. In 1871, William Hammond became the first physician to prescribe lithium for mania, specifically lithium bromide (Hammond, 1871). By the late 1890s, Li_2_CO_3_ had been used in the treatment of 35 patients with melancholic depression in Denmark (Shorter, 2009). Surprisingly, this early Danish work with Li_2_CO_3_ was seemingly forgotten, as the use of lithium in psychiatry would not experience a revival until 1949 courtesy of Australian psychiatrist John Cade (Shorter, 2009).

### 4.3 The rise of lithium carbonate

At the Bundoora Repatriation Hospital in 1949, John Cade hypothesized that excess uric acid was related to mania and summarily began treatment on 10 manic patients with lithium citrate and Li_2_CO_3_, citing the success of Garrod in using lithium to manage gout (Shorter, 2009). Cade noted that some of the patients responded quite well to the medication, with a subset of patients becoming capable of discharge from the hospital (Cade, 1949).

The timing of Cade's success was unfortunately inopportune, as in that same year, 1949, lithium chloride had failed as a replacement for sodium chloride in the management of hypertension (Corcoran et al., 1949). Reports of lithium poisoning were widespread due to the excessive use of lithium chloride‐based table salt substitutes such as Milosal and Foodsal. After several deaths, the Food and Drug Administration (FDA) banned the use of lithium salt substitutes.

Despite the concerns surrounding the use of lithium chloride, trials involving Li_2_CO_3_ use in BD continued in Australia and Denmark (Noack & Trautner, 1951; Schou et al., 1954). Interest in North America began to build in the 1960s, likely due to the advent of the Coleman flame photometer (Shorter, 2009; Coleman flame photometer, ), which allowed for the accurate tracking of blood lithium levels, and the arrival of Samuel Gershon who had previously worked with lithium at the University of Melbourne, Australia. In 1960, Gershon spearheaded the first North American publication on the use of lithium in mania (Gershon & Yuwiler, 1960). By the late 1960s, numerous investigations into the use of Li_2_CO_3_ in the management of BD had been conducted (Bunney et al., 1968; Goodwin et al., 1969; Stokes et al., 1971; Wharton & Fieve, 1966), with compelling success. In 1970, the FDA approved lithium for use in the treatment of acute mania in the United States (Shorter, 2009).

From these humble beginnings, lithium has gone on to become the arguable gold standard for the management of the manic component of BD and the prevention of mood episode recurrences (Tondo et al., 2019). However, one important question becomes immediately apparent: Why Li_2_CO_3_? A plethora of salt formulations exist, so why has Li_2_CO_3_ prescription become the norm? The answer may lie in the history of lithium as discussed above. The seminal work of John Cade concerning Li_2_CO_3_ (Cade, 1949) influenced much of the 1960s' research that led to the acceptance of lithium as a treatment option for BD (Gershon & Yuwiler, 1960), while alternative lithium salts, such as lithium chloride, were more famously involved in cases of lithium poisoning. In addition, early research was more focused on the general efficacy of lithium in the treatment of mania, less so on the differences between salt formulations. It is therefore possible that a confluence of factors pushed Li_2_CO_3_ to prominence over other salts: (1) original safety concerns surrounding alternative compounds such as lithium chloride, (2) Li_2_CO_3_ being the first and subsequently most studied formulation for psychiatric use, and (3) Li_2_CO_3_ being the first commercially produced salt for prescription.

## 5 THE PROBLEM WITH LITHIUM CARBONATE

**FIGURE 2:**
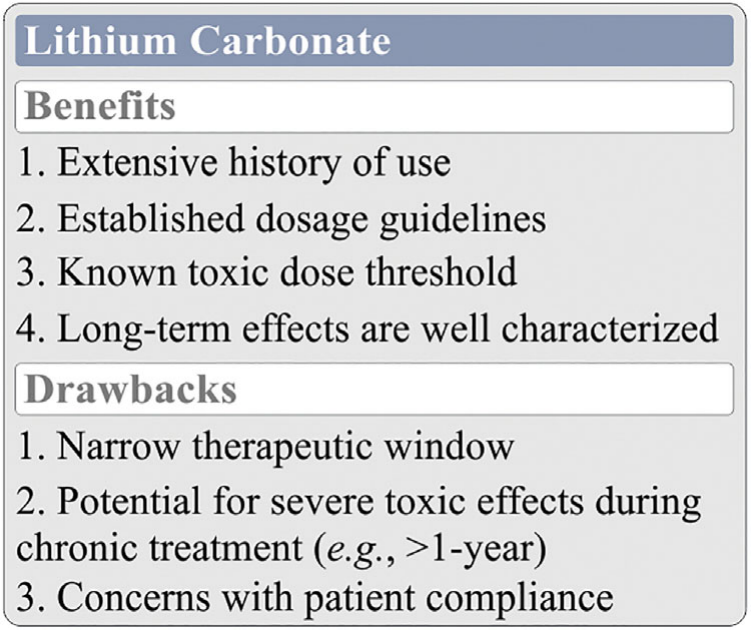
The benefits and drawbacks of Li_2_CO_3_ use in BD. Li_2_CO_3_ is a thoroughly studied compound in the management of BD. Dosage guidelines are well established, and both short‐ and long‐term toxicity concerns are well understood. Despite being the current gold standard for the control of mania and the prevention of mood episode recurrences, Li_2_CO_3_ is not without its share of issues, namely a narrow therapeutic window and a potentially severe toxic effect profile that may limit patient compliance

While incredibly effective for the prevention of manic‐ and/or depressive‐episode recurrence, Li_2_CO_3_ is not without its share of issues (Figure 2). Li_2_CO_3_, much like the other lithium salts currently approved for use, has a narrow therapeutic window, which when administered chronically can lead to very real toxicity concerns within the effective dosage range (Tondo et al., 2019). These concerns and the problems they elicit are discussed in the sections below.

### 5.1 Poor patient compliance and a significant side‐effect profile

Li_2_CO_3_, which dissociates readily in solution, requires administration of doses large enough to force elemental lithium/the lithium ion (Li^+^) across the blood–brain barrier (BBB) and into cells via sodium transporters such as the sodium‐hydrogen antiporter (NHE) (Ennis et al., 1996; Luo et al., 2018), and/or simple diffusion. The main issues with this treatment are two‐fold: (1) the dose required results in serum Li^+^ concentration that may result in significant toxicity (Gitlin, 2016; Timmer & Sands, 1999), which (2) contributes to patient non‐adherence on account of the unpleasant side‐effect profile (Jawad et al., 2018).

### 5.2 Thyroid dysfunction and renal impairment

Side‐effects of short‐term Li_2_CO_3_ use commonly involve polydipsia, polyuria, nausea, diarrhea, and tremors. More severe side‐effects, including cognitive impairment, nephrotoxicity, hypothyroidism and hyperparathyroidism (Gitlin, 2016), are typically observed during long‐term treatment, that is, 10 or more years. End‐stage renal failure is a possible, but largely unlikely outcome of long‐term lithium therapy (>10–15 years) (Malhi et al., 2012). While complications such as hypothyroidism can be managed via thyroid supplementation, this is not a desirable outcome.

The more dangerous side‐effects of lithium are elicited because the drug (1) decreases thyroid hormone synthesis and release, leading to elevated serum thyroid stimulating hormone (TSH) (Gitlin, 2016; Kibirige et al., 2013), (2) increases parathyroid hormone (PTH) release through the interruption of parathyroid cell calcium‐sensing mechanisms (Seely et al., 1989; Shapiro & Davis, 2015), and (3) disrupts antidiuretic hormone actions on the collecting ducts of the nephron, with long‐term effects culminating in nephron atrophy and interstitial fibrosis (Gitlin, 2016).

## 6 ALTERNATIVE LITHIUM SALT FORMULATIONS

The carbonate‐based salt formulation of lithium is among the mainstay treatment options for the prevention of mood‐episode recurrences, but are alternative lithium salt compounds similarly efficacious? Early in its use in medical applications, lithium was administered via a variety of different formulations, such as lithium bromide (Hammond, 1871) and lithium citrate (Fyro, 1975). When coupled with the narrow therapeutic window of Li_2_CO_3_, the very existence of alternative lithium salt compounds demands further exploration of their efficacy and safety. Might an underexplored lithium compound exist that is superior to Li_2_CO_3_?

### 6.1 Pharmacokinetics of Li_2_CO_3_: A brief overview

Li_2_CO_3_ is highly bioavailable following oral administration, with an estimated 80–100% of the ingested dose absorbed through the upper intestinal tract (Ward et al., 1994). Li_2_CO_3_ displays a relatively low volume of distribution (Mason et al., 1978), and its cellular influx and efflux appears to be driven by a combination of sodium‐dependent passive and active transport mechanisms; in general, as sodium goes, so too does lithium (Luo et al., 2018; Ward et al., 1994). Lithium is not metabolized, displays an elimination half‐life of 16–30 h, and is primarily excreted through the kidneys (Ward et al., 1994). Li_2_CO_3_ is typically delivered multiple times per day in the form of 150–600 mg tablets. The dose is titrated until consistent serum lithium levels of 0.6–1.2 mEq/L are reached; serum Li^+^ concentrations >1.5 mEq/L are avoided due to toxicity concerns (Chokhawala et al., 2020). The pharmacokinetics and pharmacodynamics of the lithium salts and organic compounds discussed in the following sections are contrasted to the above information pertaining to Li_2_CO_3_.

### 6.2 Lithium citrate

Lithium citrate, available in the commercial formulation Litarex, was approved for use in the late 1970s (Fyro, 1975). Studies exploring its efficacy have reported a range of different outcomes, from no difference relative to Li_2_CO_3_ (Guelen et al., 1992; Shelley & Silverstone, 1986), to significant reductions in peak serum lithium levels (Tyrer et al., 1982). No major differences in side‐effect profile have been noted (Sachs et al., 2000; Shelley & Silverstone, 1986). While currently—though infrequently—employed in the treatment of BD (largely in patients who have trouble swallowing caplets), lithium citrate does not appear to offer any additional benefit over Li_2_CO_3_.

### 6.3 Lithium chloride

While approved for use in BD, lithium chloride is used rarely, if ever, relative to Li_2_CO_3_. Lithium chloride sees much of its use occur in animal studies, largely due to ease of dosing via intraperitoneal injection. A study by Morrison et al. in 1971 found that when administered orally in rats, both lithium chloride and Li_2_CO_3_ displayed similar absorption and elimination patterns, with Li_2_CO_3_ resulting in generally higher plasma lithium levels at each tested concentration (Morrison et al., 2016). Given the minor differences in pharmacokinetics between the two compounds, as well as the limited shelf life of lithium chloride, owing to its hygroscopicity, there does not seem to be any reason to consider lithium chloride for use over traditional Li_2_CO_3_ preparations.

### 6.4 Lithium aspartate

Lithium aspartate, the salt of aspartic acid and lithium, is not approved by the FDA for use in the treatment of BD. At present, literature exploring its efficacy and/or safety is sparse. Select studies have investigated its potential in the cessation of alcohol abuse (Olbrich et al., 1991) and drug dependence (Dunderer, 1982), but little to no effects were noted. Further, no studies comparing the pharmacokinetics of lithium aspartate and Li_2_CO_3_ have been conducted thus far. More data are needed before any conclusions can be reached regarding the efficacy and safety of lithium aspartate relative to Li_2_CO_3_.

### 6.5 Lithium sulfate

Similar to lithium chloride, lithium sulfate is approved for use in the management of BD, but is prescribed far less frequently than Li_2_CO_3_. Studies comparing the pharmacokinetics of lithium sulfate and Li_2_CO_3_ have found little to no difference in absorption and/or peak serum lithium concentrations (Persson, 1977), or induction of side‐effects (Edstrom & Persson, 1977). There does not appear to be any reason to encourage use of lithium sulfate over the more thoroughly researched Li_2_CO_3_.

### 6.6 Lithium oxybutyrate

Research into the use of lithium oxybutyrate in BD is very limited, though a study by Liubimov et al. in 1980 demonstrated antimanic potential. In addition, the group noted that lithium oxybutyrate may potentially be more effective and less toxic than Li_2_CO_3_ (Liubimov et al., 1980). Others have found an antiarrhythmic effect in lithium oxybutyrate, which may suggest beneficial effects of the medication beyond just the control of BD symptoms (Petrova et al., 1987). The pharmacokinetics of lithium oxybutyrate relative to Li_2_CO_3_ has not been explored. Considering the promise displayed and the overall lack of investigation into its efficacy, further exploration into lithium oxybutyrate is warranted.

### 6.7 The organic anions: Salicylate, lactate, and orotate

In 2014, a study conducted by Smith et al. explored the pharmacokinetics of lithium salicylate and lithium lactate. They noted that both the lactate and salicylate formulations displayed reduced bioavailability relative to Li_2_CO_3_. However, despite lesser bioavailability, the authors proposed that lithium salicylate may represent a safer alternative to Li_2_CO_3_ on account of its more stable absorption and excretion pattern characterized by lower peak Li^+^ levels with an elongated plateau for serum concentration (Smith et al., 2014). It has previously been suggested that lithium compounds with less severe Li^+^ concentration spikes and smoother blood level curves could reduce the toxicity of lithium therapeutics (Lippmann & Evans, 1983). In fact, this is one of the arguments supporting a) the use of sustained‐release Li_2_CO_3_ formulations, as well as b) the administration of multiple daily doses (less Li_2_CO_3_ ingested per dose) in place of bulk dosing (Chokhawala et al., 2020; Couffignal et al., 2021).

LiOr represents, perhaps the most promising and controversial alternative lithium formulation. LiOr was successfully employed in the treatment of mania in the early 1970s by Hans Nieper, an early proponent for the use of orotic acid as a mineral carrier (Nieper, 1970, 1973). Contrasting studies performed in 1978 and 1979 yielded two important findings: (1) LiOr yielded significantly greater concentrations of the lithium ion (Li^+^) in the brain than was observed for equivalent doses of Li_2_CO_3_ (Kling et al., 1978), and (2) LiOr impaired renal function to a greater extent than Li_2_CO_3_ when employed at concentrations of 2 mmol Li^+^/kg body weight. The adverse effects on the kidneys reported by Smith et al. (1978) effectively closed the gates on future inquiries into the use of LiOr. While the caution advised is understandable, further research into the effects of LiOr on both kidney function and serum/brain Li^+^ levels must be conducted across a range of concentrations before LiOr can be ruled out for use in psychiatric applications; after all, the doses employed by both Kling and Smith were quite high (Kling et al., 1978; Smith & Schou, 1979).

Given the assertion that LiOr results in greater serum concentrations of Li^+^ than Li_2_CO_3_, it is entirely possible that LiOr could result in therapeutically relevant concentrations of Li^+^ at markedly reduced concentrations relative to what are typically employed during Li_2_CO_3_ therapy. Such reduced dosage requirements may alleviate the deleterious effects on the kidney noted in the 1979 study (Smith & Schou, 1979). Further work is not only warranted, but imperative to understanding the potential benefits of LiOr.

## 7 Lithium orotate: a superior lithium compound?

### 7.1 The history of orotic acid and its relevance to human biology

Orotic acid is a carboxylic acid and a pyrimidinedione, that was first discovered in whey, a proteinaceous liquid component of milk, by researchers Biscaro and Belloni in 1905 (Loffler et al., 2016). Historically, this compound was believed to be part of the vitamin B complex after it was determined in the 1950s that the previously unidentified growth enhancement factor vitamin B13, first isolated from distiller's dried grains with solubles (i.e., dried animal feed) in the late 1940s (Novak & Hauge, 1948), was in fact orotic acid (Manna & Hauge, 1953). During this period, orotic acid was identified as a precursor of pyrimidine biosynthesis (Bergström et al., 1949; Smith & Baker, 1959), highlighting its essential role in mammalian metabolism. While it is now known that orotic acid is not a vitamin, its previous status as B13 provides insight into its historical importance as a dietary component.

The primary biological role of orotic acid is to serve as a key substrate in de novo synthesis of pyrimidines (Evans & Guy, 2004). During de novo synthesis, dihydroorotate is oxidized to orotate by dihydroorotate dehydrogenase, an integral membrane protein located in the inner mitochondrial membrane with its active site oriented toward the inner membrane space. Next, orotate phosphoribosyltransferase— a component of the bifunctional uridine monophosphate synthase protein complex —catalyzes the transfer of ribose‐5‐phosphate‐1‐pyrophosphate to orotate to form the nucleotide orotidine 5′‐monophosphate (OMP). OMP is used to form uridine and other associated pyrimidine nucleotides through a series of subsequent steps and intermediated transition states (Evans & Guy, 2004; Lieberman et al., 1955).

It has also been suggested that orotic acid may exert effects beyond just the promotion of DNA/RNA synthesis through the actions of its downstream metabolites. For example, some have noted that orotic acid could increase β‐alanine pools as a consequence of upregulated uridine synthesis and subsequent metabolism (Aonuma et al., 1969; McCarty & DiNicolantonio, 2014). β‐alanine is a rate‐limiting precursor in the synthesis of carnosine (Boldyrev et al., 2013) which is a known antioxidant agent (Kohen et al., 1988,1991; Pavlov et al., 1993). Thus, endogenous and dietary orotic acids may confer cytoprotection via downstream increases in the antioxidants β‐alanine and carnosine (McCarty & DiNicolantonio, 2014). Furthermore, uridine itself serves many purposes outside of DNA and RNA synthesis; it is involved in the synthesis of various membrane components, participates in glycosylation reactions, and contributes to neuronal and glial communication as a signaling molecule recognized by purinergic‐2Y (P2Y; i.e., G‐protein‐coupled uridine triphosphate receptor) receptors (Dobolyi et al., 2011; von Kugelgen & Hoffmann, 2016). Dietary uridine, in combination with other factors such as docosahexaenoic acid, has been found to promote synapse formation in neuronal cells (Pooler et al., 2005; Wurtman, 2014), improve learning (Holguin et al., 2008), and elicit antidepressant‐like effects (Carlezon et al., 2005) in animal models.

In sum, orotic acid appears to serve many roles in in the human body, both directly, through its contributions to DNA/RNA synthesis, and (potentially) indirectly, through its downstream metabolites uridine, β‐alanine, and carnosine. While speculative in nature, the putative benefits conferred by orotic acid and its metabolites may offer an additional boon to LiOr that is absent in Li_2_CO_3_.

### 7.2 The use of orotic acid as a mineral carrier

Orotic acid‐based mineral salts were presented by Nieper in the 1970s, who noted success in the use of calcium diorotate and LiOr in the recalcification of bone metastases (Nieper, 1970) and psychiatric applications (Nieper, 1973), respectively. More recently, orotic acid has demonstrated success as a carrier for magnesium in the treatment of congestive heart failure and associated conditions in humans (Branea et al., 1999; Stepura & Martynow, 2009), and ischemia‐reperfusion injury in animal models (Mirica et al., 2013). Intriguingly, the cardioprotective and antihypertensive effects of this salt may be primarily attributable to orotic acid (Hassani et al., 2019; Rosenfeldt et al., 1998; Rosenfeldt, 1998), that may suggest beneficial effects of the chemical which are separate from its role as a carrier. Orotic acid has been proposed to improve the function of infarcted hearts by correcting adenine, cytidine, and uridine deficiencies in the stressed myocardium (Rosenfeldt et al., 1998).

While the existing literature suggests that orotate salts present an efficacious means by which to deliver minerals, modern research is lacking. Studies on both the potential efficacy of orotic acid as a mineral carrier and its mechanisms of action in accomplishing said goal of mineral delivery are needed.

### 7.3 Early interest in orotic acid as a carrier for lithium (lithium orotate)

LiOr is the salt of orotic acid and lithium. As discussed previously, LiOr and various other orotate complexes—such as, calcium diorotate (Nieper, 1970)—were presented in the early 1970s as a superior means of mineral delivery by Hans Nieper (Nieper, 1970, 1973). He proposed that orotates cross biological membranes and enter cells more readily than traditionally employed salt formulations. Despite early success marked by clinical efficacy (Nieper, 1973) in humans, and an increase in brain lithium levels relative to Li_2_CO_3_ demonstrated in animal studies (Kling et al., 1978), research into LiOr was effectively curtailed following concerns regarding potential renal toxicity in 1979 (Smith & Schou, 1979).

Interestingly, LiOr is once again attracting attention (Dell'Osso et al., 2016; Devadason, 2018; Lakhan & Vieira, 2008) for its putative ability to yield higher serum and brain Li^+^ concentrations than observed from equivalent doses of Li_2_CO_3_ (Kling et al., 1978). A recent editorial penned by Peter Devadason (2018) effectively highlights the growing interest in LiOr as a medication delivered in trace and/or supplemental dosages. The author notes the deleterious impacts of inadequate lithium intake, the reported benefits of LiOr administration, and the general lack of safety concerns associated with low‐dose LiOr supplementation (Devadason, 2018). Given the known issues associated with Li_2_CO_3_ use, perhaps it is time to explore LiOr in greater detail.

### 7.4 Can lithium orotate enter the CNS more readily than lithium carbonate?

#### 7.4.1 Potential for enhanced passage across biological membranes

**FIGURE 3:**
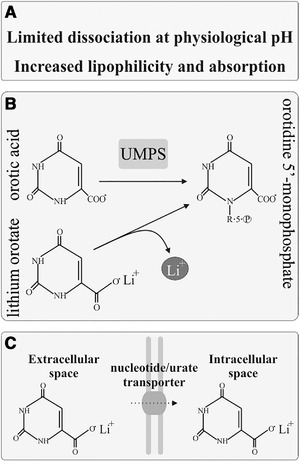
Proposed mechanisms underlying the efficacy of orotic acid as a mineral carrier. (a) Mineral orotates may display limited dissociation in biological solutions, allowing them to exist in sera as non‐dissociated, electrically neutral compounds. (b) It has been suggested that LiOr preferentially dissociates in the intracellular compartment, perhaps through mechanisms involving the uptake of orotic acid into the de novo pyrimidine synthesis pathway. (c) Given its structural similarity to non‐charged pyrimidines, LiOr may be able to make use of nucleotide transporters for passage across cell membranes, as is observed for non‐charged pyrimidines such as fluorouracil

Proponents of LiOr argue that orotates do not dissociate at physiological pH and thus exist in sera as electrically neutral compounds (Figure 3a). This is perhaps best evidenced by magnesium orotate dihydrate, which is poorly soluble in water and is known to not bind gastric acid. Further, in contrast to the more easily dissociable magnesium salts, magnesium orotate dihydrate does not exhibit a laxative effect following oral administration (Classen, 2004).

A neutral, non‐dissociated LiOr complex could potentially cross the BBB and cell membranes with greater efficiency than the Li^+^ and carbonate ions of Li_2_CO_3_ (Nieper, 1973; Sartori, 1986), which dissociate readily in solution. Also, as orotate is a precursor to uracil, orotates may make use of nucleotide transporters (e.g., the uracil transporter) resident in cell membranes (Figure 3c). Non‐ionized fluorouracil is a substrate for such transporters, while charged pyrimidines are not (Wohlhueter et al., 1980), which suggests neutral orotate complexes may also be substrates. Finally, orotate is known to utilize the urate transporter 1 (URAT1) for traversal across membranes in the kidney (Miura et al., 2011), which may suggest that passage into the CNS is possible via URAT1 located within the choroid plexus (Chiba et al., 2020; Uemura et al., 2017).

#### 7.4.2 Putative target specificity of lithium orotate may involve the pyrimidine synthesis pathway

Orotate is an intermediate in pyrimidine biosynthesis, which occurs intracellularly (Evans & Guy, 2004; Lieberman et al., 1955). Hans Nieper proposed that orotates demonstrate affinity for tissues where metabolism heavily features the pentose phosphate pathway (PPP) and subsequent pyrimidine synthesis, such as glia, vascular endothelium (BBB in particular), and neurons (Nieper, 1973) (Figure 3b). Increased neuronal PPP has been suggested as a means to maintain redox homeostasis in highly metabolically active tissues (Stincone et al., 2015). Lithium may be liberated from the orotate carrier within the cell during the incorporation of orotate into the synthesis pathway, allowing for enhanced intracellular accumulation of Li^+^ relative to Li_2_CO_3_, which dissociates extracellularly. Increased intracellular accumulation of Li^+^ may in part explain why LiOr results in greater long‐term (i.e., >24 h) brain Li^+^ concentrations than what are observed for equivalent doses of Li_2_CO_3_ post‐administration (Kling et al., 1978).

### 7.5 Potential concerns regarding use of lithium orotate

#### 7.5.1 Impaired renal function

LiOr received a brief surge of interest in the early‐to‐mid 1970s in part because of advocacy from Nieper, who argued for the use of orotate as a mineral transporter (Nieper, 1970, 1973). This was predicated on the fact that mineral orotates, including LiOr, do not dissociate at physiological pH, and may thus diffuse across biological membranes more readily on account of their electrically neutral state. Interest was greatly curtailed in the late 1970s after Smith et al. noted that LiOr resulted in reduced glomerular filtration rates relative to Li_2_CO_3_— though the mechanisms underlying these effects are unknown and may be dose‐dependent —and advised against its consideration as a treatment option (Smith & Schou, 1979); however, the study in question employed equivalent high concentrations of LiOr and Li_2_CO_3_, thereby defeating the suggested purpose of administering LiOr in place of Li_2_CO_3_.

It should be noted that the putative transport capacities of the orotic acid carrier could potentially contribute to worsened organ toxicity. If the increased ease of membrane transport associated with LiOr compared to Li_2_CO_3_ is not unique to the BBB and/or elements of the CNS, then the elevated accumulation of lithium within off‐target organs could accelerate development/risk of complications, for example, renal dysfunction.

With that being said, there have been no reported cases of death or serious side‐effects in over 40 years of use in North America (Devadason, 2018). In fact, one of the more well‐known cases of LiOr toxicity appears to highlight the safety of the compound. In 2007, Pauzé and Brooks submitted a case report for a patient who had ingested 18 LiOr tablets, with each tablet containing 3.83 mg of Li^+^. The patient displayed nausea, one episode of emesis, minor tremors, and normal vital signs; all symptoms resolved after 3 h of observation with no intervention (Pauzé & Brooks, 2007).

#### 7.5.2 Promotion of cancerous cell growth

Dietary orotic acid has been linked to the promotion of hepatocarcinogenesis in rats acutely exposed to known carcinogens (Laconi et al., 1993; Laurier et al., 1984). While a cause for concern, there are a few factors worthy of note: (1) Stimulated cancerous cell growth was only observed in initiated rats; that is, rats destined for hepatocarcinoma. (2) The amount of orotic acid ingested in the course of LiOr therapy by BD patients (i.e., <1000 mg or ∼10–15 mg/kg) is unlikely to contribute to the growth of cancerous cells, as stimulation of cancerous cell growth was observed at orotic acid concentrations exceeding 100 mg/kg (Laconi et al., 1993). Nevertheless, use of orotic acid formulations, be it for lithium, magnesium, iron, or copper, is potentially not advisable for individuals known to be at risk for oncogenesis.

### 7.6 Evidence supporting the efficacy of lithium orotate

#### 7.6.1 Animal studies

Animal studies exploring LiOr are limited. In 1976, Donald Smith examined the pharmacokinetics of LiOr, Li_2_CO_3_ and lithium chloride, noting minimal differences between the compounds (Smith, 1976). Conversely, a study conducted in 1978 by Kling et al. noted that rats injected intraperitoneally with either 1, 2 or 4 mmol Li^+^/kg of body weight, displayed markedly higher brain and serum concentrations of Li^+^ at each dose when Li^+^ was delivered as an orotate instead of as a carbonate (Kling et al., 1978). Furthermore, when administered at 2 mmol Li^+^/kg as an orotate, Li^+^ progressively accumulated within the brain in a manner not observed for Li_2_CO_3_; Li_2_CO_3_ maintained a steady concentration of 0.5 mmol Li^+^/kg of brain tissue, whereas LiOr increased from 0.5 to ∼1.3 mmol Li^+^/kg over the course of 24 h. Brain lithium concentrations were assayed via flame photometry of tissue homogenates.

Shortly thereafter, in 1979, Smith and Schou reported a potential for increased risk of renal toxicity for LiOr relative to Li_2_CO_3_ in rats when injected at high concentrations, that is, 2 mM Li^+^ (Smith & Schou, 1979). As Kling et al. (1978) observed that LiOr maintained higher 24‐h serum concentrations than Li_2_CO_3_, it is possible that LiOr displays heightened brain and serum Li^+^ levels post‐administration due to reduced renal clearance, an argument put forth by Smith and Schou (1979). Thus, studies examining the membrane transport mechanisms of LiOr, and any connections they may have to the reported elevations in serum and brain Li^+^ content relative to Li_2_CO_3_, are needed.

To date, these studies appear to be the only cases directly contrasting LiOr and Li_2_CO_3_. Comparisons of the pharmacokinetics, safety, and efficacy of LiOr and Li_2_CO_3_ are sorely lacking in the literature.

#### 7.6.2 Clinical studies

Clinical studies exploring the efficacy of LiOr are sparse, perhaps as a result of the study by Smith and Schou (1979) discussed previously. While no clinical trials for the use of LiOr in BD have been conducted, LiOr has shown success in promoting cessation of alcohol abuse when administered daily at 150 mg for 6 months; out of the 42 patients involved, 23 remained without relapse for 1–10 years (Sartori, 1986). While LiOr effectively reduced relapse occurrence at the relatively low dose of 150 mg (Sartori, 1986), Li_2_CO_3_ demonstrates only mild efficacy at therapeutic dosages, that is, >600 mg (Fawcett et al., 1987). Furthermore, other studies have shown no effect of Li_2_CO_3_ on avoidance of relapse (Dorus et al., 1989), leading some to conclude that Li_2_CO_3_ is not an effective treatment for alcoholism (Lejoyeux & Ades, 1993). No additional studies concerning the use of LiOr in the treatment of alcoholism have been conducted outside of the work by Sartori (1986). In sum, LiOr may reduce the occurrence of relapse in alcoholism at subtherapeutic doses, while Li_2_CO_3_ does not appear to be similarly effective even at markedly higher concentrations, lending credence to the notion that LiOr may be a more efficacious formulation.

## 8 CLOSING REMARKS: THE FUTURE OF LITHIUM OROTATE

**FIGURE 4:**
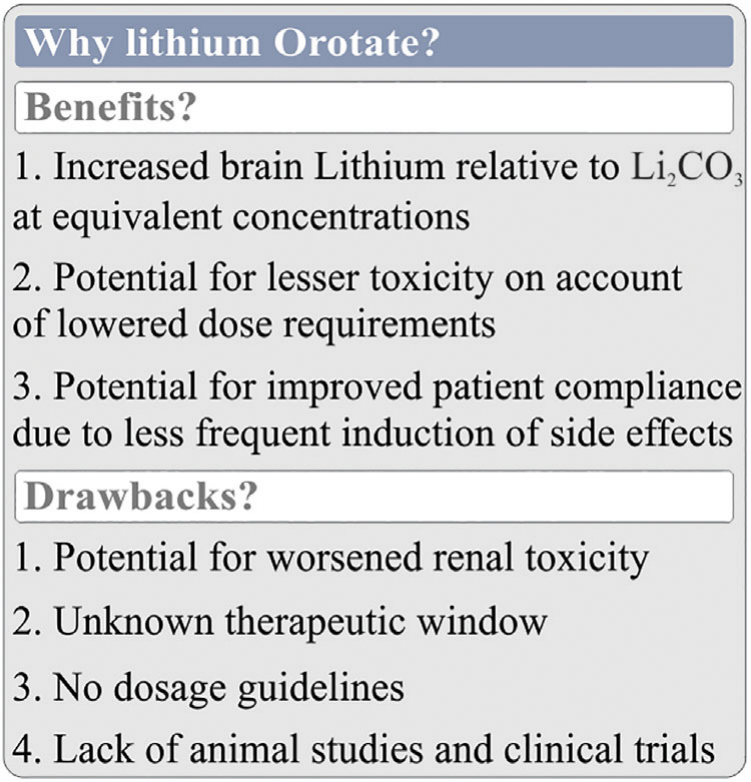
LiOr as a replacement for Li_2_CO_3_ in the treatment of BD. Proponents of LiOr argue that LiOr can cross biological membranes and enter cells more readily than Li_2_CO_3_, allowing for lesser concentrations to be administered while maintaining an equivalent therapeutic effect. While LiOr has been found to result in higher brain concentrations of lithium than Li_2_CO_3_, others have noted that this may come at the cost of increased renal toxicity. More research into both benefits (e.g., increased accumulation within cells) and drawbacks (e.g., renal toxicity) is needed

There is one certainty regarding the future of LiOr in psychiatric applications: more research is needed. Orotic acid has shown promise as a mineral carrier for calcium (Nieper, 1970), magnesium (Bambling et al., 2017; Classen, 2004; Mirica et al., 2013; Stepura & Martynow, 2009), and lithium (Nieper, 1973; Kling et al., 1978; Sartori, 1986); however, concerns regarding its toxicity have also been noted (Laconi et al., 1993; Laurier et al., 1984; Smith & Schou, 1979). In short, while the potential of lithium orotate in the management of BD symptomatology is fascinating, clinical application is not recommended at this time due to the scarcity of literature concerning its benefits and risks (Figure 4).

Before LiOr can be seriously considered as an alternative to Li_2_CO_3_, studies exploring its efficacy in both basic science and clinical settings need to be conducted. This need is especially pressing given the nutraceutical status of LiOr, and the consequent wide‐spread use of the compound within the self‐medicating population. Furthermore, some physicians and alternative health practitioners recommend small doses of LiOr to their patients, plausibly for its mood stabilizing properties at such concentrations (Devadason, 2018). While these small doses are likely to be safe, the potential toxic effects of micro‐dosing LiOr still warrants further examination. However, it is reassuring that daily intake of 150 mg of LiOr over 6‐months during an alcohol‐abuse cessation study resulted in only minor adverse effects in 8 out of 42 patients (Sartori, 1986), and even excessive use of LiOr tablets (18 total) failed to elicit a severe acute adverse reaction in a case report presented by Pauze and Brooks (2007).

Ideally, exploratory experiments into the efficacy, toxicity, and membrane transport mechanisms of LiOr will be conducted in animal models before any further human clinical trials are considered. First, the putative ability of LiOr to enter cells more readily than Li_2_CO_3_ should be examined. Does LiOr truly result in higher brain and serum concentrations of lithium relative to Li_2_CO_3_, as was previously observed by Kling et al. (1978)? If so, how does the LiOr complex accomplish this? What is/are the mechanism(s) of action by which this is achieved? In vivo imaging of lithium distribution in the CNS via ^7^Li magnetic resonance (NMR) following LiOr and Li_2_CO_3_ administration may address some of these questions (Smith et al., 2018; Stout et al., 2020).

Second, it must be determined whether the ease of membrane transmission proposed for LiOr is unique to the BBB, or if organs such as the kidney or thyroid are equally susceptible to accumulation. If they are, then the enhanced membrane transport capacities of orotic acid may still result in toxicity despite reduced dosage requirements. Ex vivo (^7^Li NMR) or in vitro (colorimetric or photoelectric assay of tissue homogenates) assessment of lithium accumulation within various target organs, such as the brain, thyroid or kidney, would effectively address such concerns.

Third, the effects of orotic acid itself should be characterized, especially in light of the potential positive actions mediated by the downstream metabolites carnosine and uridine (Aonuma et al., 1969; Dobolyi et al., 2011; Holguin et al., 2008; McCarty & DiNicolantonio, 2014), as well as the concerns regarding the promotion of oncogenesis (Laconi et al., 1993; Laurier et al., 1984, Laconi et al., 1993). While the risk of oncogenic effects appears only to be significant at excessive concentrations (Laconi et al., 1993; Laconi et al., 1993), such risks should nonetheless be examined using whole animal‐ and cellular model‐based studies in which therapeutically relevant concentrations of LiOr are employed.

Finally, short‐term and long‐term toxicity studies must be performed to address the legitimate concerns raised by Smith and Schou (1979), especially should the enhanced membrane transport capacities of LiOr prove to extend to organs other than the brain.

## CONFLICT OF INTEREST

The authors have no conflict of interest to declare.

## AUTHOR CONTRIBUTIONS

*Conception of central ideas and editing*: Lane K. Bekar and Anthony G. Pacholko. *Writing*: Anthony G. Pacholko.

All named authors have seen and approved the final version of the manuscript.

### PEER REVIEW

The peer review history for this article is available at https://publons.com/publon/10.1002/brb3.2262.
